# Complete Genome Sequence of Chikungunya Virus Isolated from an *Aedes aegypti* Mosquito during an Outbreak in Yemen, 2011

**DOI:** 10.1128/genomeA.00789-15

**Published:** 2015-07-16

**Authors:** Nermeen T. Fahmy, John D. Klena, Amr S. Mohamed, Alia Zayed, Jeffrey T. Villinski

**Affiliations:** a United States Naval Medical Research Unit No. 3, Cairo, Egypt; b Global Disease Detection Branch, Division of Global Health Protection, United States Centers for Disease Control and Prevention, Atlanta, Georgia, USA; c Faculty of Science, Cairo University, Cairo, Egypt

## Abstract

Chikungunya virus is recognized as a serious public health problem. The complete genome was sequenced for a chikungunya virus isolated from the mosquito *Aedes aegypti* during a 2011 outbreak in Al Hodayda, Yemen, which resulted in significant human fatalities. Phylogenetic analysis demonstrated that this Yemeni isolate is most closely related to Indian Ocean strains of the east/central/south African genotype.

## GENOME ANNOUNCEMENT

Chikungunya virus (CHIKV) is an enveloped, positive-sense, single-stranded RNA arthropod-borne virus, which is taxonomically classified as a member of the *Alphavirus* genus, family *Togaviridae* (1). CHIKV is transmitted to humans via mosquitoes of the genus *Aedes*, with *Aedes aegypti* and *Aedes albopictus* being the primary vectors. In January 2011, the World Health Organization (WHO) detected an outbreak of a dengue-like illness in Al-Hodayda, Yemen. Initially, 1,542 cases were reported, with 104 associated deaths (case fatality rate, 6.5%) (2). Mosquito samples collected by our institution from the outbreak focus tested positive for CHIKV (2). We report here the complete genome sequence for a CHIKV isolate recovered from an *A. aegypti* female mosquito (head and thorax samples) collected from an indoor environment during the 2011 Yemen outbreak.

CHIKV was isolated by cell culture (2). RNA was extracted from the culture supernatant using the QIAamp viral RNA extraction kit (Qiagen, Valencia, CA). cDNA amplification and sequencing were performed as previously described (3) using a BigDye Terminator version 3.1 cycle sequencing protocol and the 3130xl genetic analyzer (Applied Biosystems, Foster City, CA). The whole-genome sequence was assembled using BioEdit (version 7.0) and submitted through the NCBI BankIt tool. Percent identity and similarity among the nucleotides and amino acid sequence were calculated between this Yemeni isolate and reference sequences. The Yemeni CHIKV genome is 11,778 nucleotides (nt) long, and two open reading frames of 7,413 nt and 3,747 nt were identified, encoding nonstructural and structural polyproteins, respectively.

Whole-genome analysis reveals the Yemeni CHIKV isolate is 99.5% similar to the Indian Oceans strains but only 96.7% similar to the S27 African CHIKV prototype (GenBank accession no. NC_004162). The Yemeni isolate has several unique amino acid substitutions in the nonstructural and structural protein regions: two in the nsP2 region (I447M and N667Y), 5 amino acid deletions in the nsP3 at positions 368 to 372, one in the nsP4 region (L385H), and one in the E2 region (V251A). A deletion observed in the nsP3 region is of particular interest, as this locus is involved in negative-strand RNA synthesis, a function critical to the virus. The biological effect, if any, of this deletion should be determined.

Phylogenetic trees were generated using MEGA (version 6.0) (4) with the neighbor-joining method. The Yemeni CHIKV strain shares high similarity with the geographically proximate Indian Ocean strains of the east/central/south African (ECSA) genotype, indicating a potentially recent divergence from this source. The Yemeni isolate does not possess the E1 (A226V) substitution, a recently evolved mutation thought to enhance transmission via a new host mosquito, *A. albopictus* (5).

### Nucleotide sequence accession number. 

The assembled complete genome sequence of the Yemeni CHIKV isolate was submitted to GenBank under the accession no. KC614648. The version described in this paper is the first version.
